# Supplementary material for: Identification of photoperiod-induced specific miRNAs in the adrenal glands of Sunite sheep (Ovis aries)
Source: Front Vet Sci. 2022 Jul 22;9:888207. doi: 10.3389/fvets.2022.888207 (PMC9354845; doi:10.3389/fvets.2022.888207)
Supplement: Supplementary file 1 [file Data_Sheet_1.ZIP › Supplementary file/miRDeep2 file.zip!/miRDeep2 file/Novel_Structure/LP42A1/Novel_522.pdf]

Provisional ID : NC_019479.2_31284
Score total : 2.1
Score for star read(s) : -1.3
Score for read counts : 0
Score for mfe : 1.8
Score for randfold : 1.6
Score for cons. seed :
Total read count : 15
Mature read count : 15
Loop read count : 0
Star read count : 0

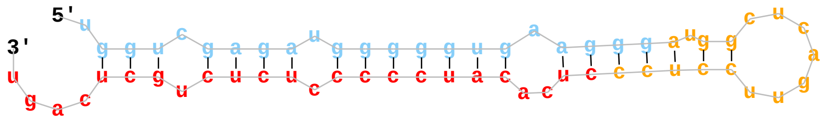

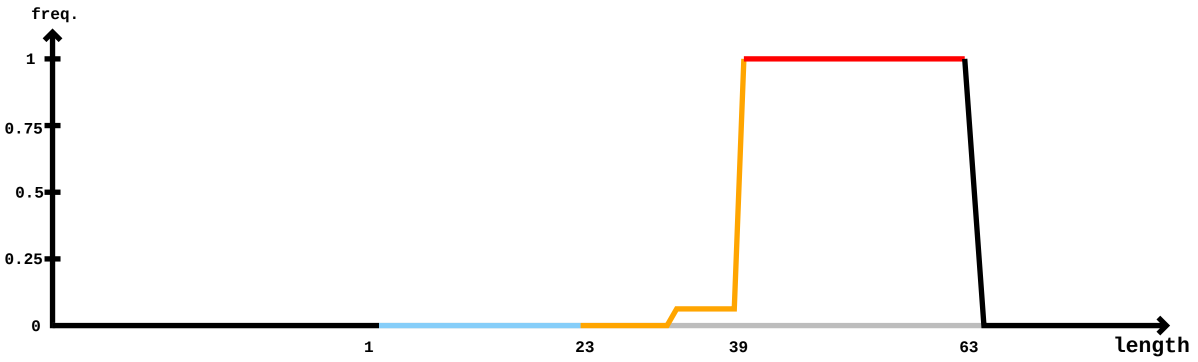

Star Mature

```
5'- uggacaucugcugucuuagauucagauggaacuggucgagaugggggugaagggauggcucaguuccucccucacaucccccucucugcucaguuaaucaggaagccaacgugc   -3'    exp
    (((.((((((..(((...))).)))))).(((((((.((((.(((((((.(((((.((.......)))))))..))))))).)))).)).)))))..........)))......    reads    mm    sample
    ...............................................................uuccucccucacaucccccucucugcucagu....................    1        0     seq
    ......................................................................cucacaucccccucucugcucagu....................    15       0     seq
```
